# Tools for assessing teacher digital literacy: a review

**DOI:** 10.1007/s40692-022-00257-5

**Published:** 2023-01-19

**Authors:** Lan Anh Thuy Nguyen, Anita Habók

**Affiliations:** 1 Doctoral School of Education, University of Szeged, Szeged, Hungary; 2 Institute of Education, MTA-SZTE Digital Learning Technologies Research Group, University of Szeged, Szeged, Hungary

**Keywords:** Digital literacy, Digital competence, Literature review, Assessment tools, Professional development, Educational assessment, Teacher evaluation

## Abstract

With the rapid advancement of technology, digital literacy has become a key component in educators’ professional development. A wide range of assessment tools has been developed to measure teacher digital literacy; however, there has been no previous attempt to systematically synthesize and scrutinize those tools to improve evaluation of this ability among educators. The current study reviews literature on instruments that assess teacher digital literacy with the purposes of ascertaining the main aspects of it that recent researchers focus on in their evaluation, instrument types used for assessment, and the reliability and validity report, as well as the frameworks or models used to design assessment tools. The review selected 33 English-language publications in the field of educational technology from peer-reviewed journals indexed in the Education Resources Information Center (ERIC), Web of Science, and Scopus. The study period spanned from 2011 to 2022 with the objective of reviewing the tools used to assess teacher digital competence. The major findings demonstrate that scholars focus on digital competence in teachers’ use of educational technology, teaching and learning, professional development, and support for learners through digital competence. Other researchers emphasize the ability of educators to apply technology to the assessment of learner outcomes or to empower students in using technology to enhance learning. Additionally, self-evaluation instruments are common, whereas a few studies promote subjective evaluation in combination with objective assessment to provide a comprehensive understanding of teacher digital competence. The results form the basis for several recommendations for future research for the further examination of teacher digital literacy.

## Introduction

There has been a great deal of effort to provide technologies in schools since the 1980s because educational computing has long been considered to prepare learners with essential digital skills for their future career. However, the issue of the integration of technology into an educational context positively facilitating the outcome of teaching and learning has been controversial because there has been a lack of evidence for the effectiveness of using technologies in education (Elstad, 2016). The concern here is that businesspeople, the technology industry and policy-makers may merely use the inclusion of digital technology as rhetoric to gain access to lucrative markets (Lindh & Nolin, 2016), while students, teachers, and teacher education are often overlooked (Nivala, 2009). Specifically, in recent years, globalization and the expansion in information and communication technology (ICT) have been used to augment the importance and urgency of implementing ICT in education. Keeping up with the times to avoid being left behind, multiple governments have invested in reformulating the education system to align it with the global information society discourse because education is understood as one of the most influential strategies to facilitate national development in the digital age (Haugsbakk, 2013). Accordingly, digital literacy development is considered an instrument to improve educational standards, address economic problems, and build the information society (Hanell, 2018). The ubiquitous involvement of technology in all aspects of education and changes in ICT policies have been demonstrated by the fact that a series of education systems have required digital educational platforms or assessment tools for learning and teaching (Porat et al., 2018). Therefore, at a high-stakes level, digital literacy development is considered one of the strongest potential solutions to the multi-faceted problems of involving ICT in society, since it can become an instrument for better educational attainment and societal development (Hanell, 2018; Nguyen & Habók, 2021). At a low-stakes level, digital literacy is also becoming a major concern for school stakeholders because investing in digital facilities to support a hybrid teaching and learning system would only be valuable if teachers and students possessed sufficient digital literacy to use technology in education effectively. In the current educational environment, teachers’ mission is to support students in mastering the knowledge and skills required in the twenty-first century. Moreover, digital literacy is considered one of the key competencies as well as an essential factor in learning (Knutsson et al., 2012) for students to be able to cope with the demands of globalization. Additionally, scholars predict that digital literacy will be indispensable in all kinds of achievement during the Fourth Industrial Revolution (Williamson et al., 2019). Educational technologies, the task of being digital role models, and providing appropriate education for students as future citizens are crucial. Thus, teachers are required to achieve high levels of qualified digital literacy or to possess sufficient technical, cognitive, and socioemotional skills (Güneş & Bahçivan, 2018). Teachers can thus successfully align technologies, pedagogies, and content knowledge in a digitally rich media environment. Additionally, in relation to coping with the digitization of education, teachers are urged to update, enhance, and emulate well-honed skills in front of students (Priestley, 2011). The reason for this notion is that teachers play a decisive role in learner digital literacy. Moreover, teachers are considered a primary factor in the success of placing and innovating technology in schools (European Union, 2013). Consequently, professional development among teachers in general and their level of digital literacy in terms of integrating technology into education in particular have become global concerns. In response, researchers around the world have conducted various studies across contexts to assess the extent to which educators use technologies in the teaching process. Moreover, such studies intend to examine the ability of teachers to guide students in the use of technology through digital literacy. As such, scholars formulate and test diverse instruments to assess teacher digital literacy in substantial educational contexts (e.g., Quaicoe & Pata, 2020). Furthermore, measurement is a complex process because of the dizzying emergence of technologies in society (Núñez-Canal et al., 2022). Scholars therefore recommend that educators constantly improve knowledge and skills for educational practice and professional development. While a variety of measurements has been presented in various studies to evaluate teacher digital literacy, there is a need to categorize and synthesize recent assessment tools to facilitate improvement of evaluation. Additionally, the existing literature focuses a great deal on reviewing digital literacy assessment among students (e.g., Siddiq et al., 2016) but not among teachers. The objective of this review is thus to paint a picture of the assessment of teacher digital literacy in the school context. The paper intends to answer the following questions:

### RQ1

What aspects of digital literacy are presented in recent research on potential ways to assess teacher digital literacy?

### RQ2

What types of instruments are used to assess digital literacy, and how are instrument reliability and validity reported?

### RQ3

What frameworks or models are used to develop the instruments?

With these guided research questions, the article will be followed by a theoretical background section where digital literacy and other related concepts, dimensions of digital literacy, models/frameworks for assessing educators’ digital literacy, approaches and types of instruments, and reliability and validity indicators for instruments are reviewed comprehensively. After that, we present the methodology with information on the coding process and study selection. In the Results and discussion section, the research questions will be answered and discussed in detail. In the final section, we will synthesize the main findings of the research and point out some limitations as well as making recommendations for future studies.

## Theoretical background

### Digital literacy and other related concepts

The unprecedented growth of ICT in the digital era has spawned various terms to indicate the skills, competencies, abilities, or literacies related to the use of digital technologies. Apart from digital literacy, which was used by the European Commission (2003), various studies have coined a wide range of other similar terms. The most common ones are ICT literacy (Educational Testing Service, 2002), twenty-first-century skills (Partnership for 21st Century Skills, 2002), new literacies (Lankshear & Knobel, 2003), digital skills (Erstad, 2006), media literacy (Erstad, 2010), digital competence (Ferrari et al., 2013), Internet literacy (Harrison, 2017), emerging technology (Pacansky-Brock, 2017), and ICT competence (Suárez-Rodríguez et al., 2018). However, no clear-cut boundaries were established between the constructs covered by these terms in previous studies. Frequently, certain authors have used related terms to replace others (e.g., Nguyen & Habók, 2022a). Thus far, the relationship between these terms has remained controversial in the literature, with the differences possibly having originated because of the multiple academic fields represented by the authors (Bawden, 2008) or the occasional expansion of the technologies. Indeed, numerous terms were used in the digital context when technologies were not as developed as at the present time, in which the context was sometimes implicit and sometimes explicit (Ala-Mutka, 2011). This is why scholars may use the same term but with different foci or different terms with the same focus (Bawden, 2001). Therefore, it is somewhat challenging to employ a term because the relevant concept may be too broad or narrow. If the concept is too broad, the findings of the study have no purpose. In contrast, the discovery may rule out significant components. However, it cannot be denied that the terms noted above share similarities because they focus on the use of technologies in coping with information and communication and with content creation through technologies to aid an individual in achieving targets in learning, professional development, and other activities (Hatlevik et al., 2015). Additionally, these terms are the links between technology domain, knowledge, competence, and ethical issues (Siddiq et al., 2016).

### Conceptualization of digital literacy

The concept of digital literacy can be encapsulated using multiple means and perspectives. Although certain concepts of digital literacy have offered a panoramic vision, others have endeavored to dissect the terminology to understand what lies at the heart of it. Initially, the definitions seemed contradictory and were defined from different perspectives and in different contexts. A more comprehensive understanding of digital literacy was constituted out of a combination of such definitions. For example, digital literacy could be captured from the cognitive perspective through expansive definitions. For example, Gilster (1997) proposed one of the earliest and most common definitions in relation to an understanding of digital literacy and described the term as the competence to use, evaluate, and align multiple digital resources or tools in the lifelong learning process. This definition exceeded the narrow scope of technical skills and emphasized the issue of generating the idea of using technology to solve problems and to acquire vital knowledge and skills in the digital context. This then formed a scaffold for later conceptions of digital literacy (e.g., Nguyen & Habók, 2022b; Redecker, 2017a), whereas the limits of the term have gradually been expanded on the basis of new skills and updated digital technologies in multiple digitized environments. Additionally, the European Commission (2018) suggested that digital literacy is a vital competence such that the accessibility and use of technologies enable individuals to achieve personal integration and development in society (European Commission, 2018). These general definitions have been applied to different fields, where the scope is then associated with additional specific contexts. In education, teacher digital literacy is related to proficiency in applying technology to education accompanied with awareness of and decisions on the implications of teaching and learning in the context of a digitized society (Krumsvik, 2012). Another example is Hall et al. (2014), who developed the DigiLit Leicester project to facilitate progress in teacher digital literacy. The working definition of the project emerged from various definitions presented by a number of education studies. Also, in the project, they defined teacher digital literacy as knowledge, skills, and attitudes toward the use of educational applications to support learners. A digitally literate teacher is expected to be capable of effectively teaching with technology, enhancing their professional development, critical thinking about technology integration, and forming their identity. The government of Catalonia also identified digitally competent educators as those who can implement educational knowledge, skills, and attitudes in concrete teaching situations on a daily basis (Generalitat de Catalunya, 2018).

Digital literacy was also found in many studies on narrow-scope definitions of digital literacy, which mainly focus on operational technological tasks (e.g., Son et al., 2017). The Assessment and Teaching of twenty-first Century Skills project concentrates on improving learners’ consumer and producer skills and social and intellectual skills in the digitized collaborative context in relation to digital literacy (Wilson et al., 2017). Reviewing the foundational definition of digital literacy and distinguishing from Gilster’s (1997) cognitive aspect, the author noted that accessibility, information search, management and editing, and virtual communication and network participation comprise a set of skills that requires digital literacy. In education, researchers have adopted the concept by classifying different technical skills related to the accessibility of information, online involvement, computer skills, search engine skills, and information evaluation (McArthur et al., 2018). This paper will focus on digital literacy among educators in a specific context, which is the educational environment. Then, once we have summarized, narrowed, and linked the definitions above, digital literacy can be understood as educators’ knowledge, skill, and attitude in dealing with technologies to facilitate teaching and learning, professional development, and other educational activities.

### Dimensions of digital literacy from different perspectives

Digital literacy has been categorized into types of subcompetencies based on hierarchy. Authors and scholars continue to discuss the term, other related constructs, and the scope of these constructs. Although Law et al. (2018) categorized digital literacy into several subcompetencies, computer, ICT, information, and media literacies, Wilson et al. (2011) classified these terms in the opposite direction. Specifically, the authors combined media and information literacies into an umbrella term that covers digital literacy. From the same perspective, Tristán-López and Ylizaliturri-Salcedo (2014) claimed that digital literacy and other concepts, such as information and computer literacies, are subcomponents of ICT competency, which is considered a blanket term. Alternatively, other authors have refrained from linking these associated constructs to establish a class relation, and this perception may be reasonable because the concepts above share similarities (e.g., Hatlevik et al., 2015), which we discussed in the previous section. However, specific sub-branches that constitute digital literacy should be determined to capture the concept fully. Digital literacy may be assumed to comprise six branches of subcognitive literacies: photo-visual (comprehension of multimedia information), reproduction (creation of a completed product from disparate information), branching (characterization, arrangement, and engagement of available information), information (critical evaluation of information), socioemotional (adherence to digital norms), and real-time thinking (simultaneous processing of a number of stimuli) literacies. Eshet (2012) proposed this classification, whereas Ng (2012) presented three broad dimensions: technical, cognitive, and social–emotional aspects. Although the concept includes the essential skills of digital literacy, scholars have voiced criticism that operational skills, which are related to the ability to work using different, updated hardware and software for specific purposes, should have been indicated (Zhong, 2011). Indeed, both views are persuasive, and they are impacted by the research contexts, the purposes of the research, the field represented by the authors, and so on. A few years later, Carretero et al. (2017) promoted a European digital competence framework for citizens: DigComp 2.1. The framework specified digital literacies on five subscales: information and data literacy, communication and collaboration, digital content creation, safety, and problem-solving. The framework has become influential in the assessment of digital literacy and has been used in multiple fields (e.g., Silva-Quiroz & Morales-Morgado, 2022). Eventually, the framework was adopted to measure digital competence among citizens across fields. Moreover, Van Laar et al. (2017) intended to assess digital literacy in an authentic, specific context. Hence, the authors specified not only digital literacy but also the contextual skills required to implement it. The core skills recommended by the authors cover seven core elements (technical, information management, communication, collaboration, creativity, critical thinking, and problem-solving). Apart from these seven elements, the authors also categorized contextual skills to facilitate the use of digital literacy in various contexts: ethical awareness, cultural awareness, flexibility, self-direction, and lifelong learning. Similarly, Peromingo and Pieterson (2018) grouped digital literacy into five components: operation, mobility, navigation, society, and creation. However, the authors added the ability to use mobile devices as a component of digital literacy apart from computers, which are common tools, due to the current popularity of such devices in the classroom environment. Clearly, there are trends in components of digital literacy which must adapt to the development of technologies and society.

### Models/frameworks of teacher digital literacy assessment in the educational context

Various empirical studies have designed and tested a number of frameworks and assessment tools that describe multiple components of digital literacy at the international and national levels. The objective was to support the measurement of teacher digital literacy to anticipate training needs (Redecker, 2017a) or to explore the extent to which these frameworks or measurement tools interpret teacher competence in the educational context.

At the international level, Martin and Grudziecki (2006) developed the DigEuLit framework and tools for European countries and proposed three levels for the enhancement of digital literacy from the foundation to the extreme stage (digital competence, use, and transformation). The framework provided educators, learners, and learning support staff with specific guidelines on how to facilitate the recognition of digital components in teaching, learning, and support activities in line with the curriculum and with professional development. Moreover, the framework emphasized the application of technology for working and learning purposes in practice. It was further tied to several tools for tutors, learners, and support staff to enable them to monitor, deliver, or acquire the appropriate digital elements for teaching and learning programs. Nevertheless, the focal points of the framework and tools are inadequate in terms of relevant skills outside schools. The rapid change in digital technologies in the new decade requires new competencies for educators and learners. New frameworks have thus been designed and developed to cope with the pace of society. Another framework has been published for the context of European education, the European Framework for the Digital Competence of Educators (DigCompEdu; Redecker, 2017b), which was modified from DigComp (Carretero et al., 2017). This framework is based on consultations among experts to describe the digital competencies necessary for the teaching profession and to identify the specific digital literacies required of educators. The framework has been widely recognized because it not only adopted relevant skills from valuable frameworks but also complemented the comprehensive aspects of the twenty first-century competencies necessary for teachers. The framework focused on multiple aspects that educators address in their daily professional activities, such as professional engagement, digital resources, teaching and learning, assessment, learner empowerment, and facilitating digital competence among learners. The core of the framework intended to support teachers in implementing technology in teaching in a pedagogically effective manner as well as to aid learners in achieving the skills required in the digital business world. Thus, the results of this stream of research could be interpreted as follows: technology, pedagogy, and target knowledge and skills should be incorporated to promote meaningful teaching in a digitized learning environment. Additionally, the core elements of the framework are not separate but united in focusing on establishing links to apply technology and pedagogy to teaching practice in relation to problem-solving skills in the real world. Moreover, UNESCO (2013) designed the ICT Competency Framework for Teachers (ICT-CFT), which was developed on the basis of three integration levels in ICT (technology literacy, knowledge deepening, and knowledge creation) to support policy-makers in assessing ICT competency among teachers. Thus, the framework aimed to measure the competency of teachers from six aspects in relation to the education system (policy and vision, curriculum and assessment, pedagogy, ICT, organization and administration, and teacher professional development) from the perspectives of curriculum designers and policy-makers. DigCompEdu and UNESCO ICT-CFT mainly focus on teachers’ application of technology to teaching activities using consistent aspects of assessment. However, UNESCO ICT-CFT is also concerned with the issue of context as regards curriculum, facilities, and policy. As such, it expects teachers to address issues associated with specific contexts to effectively integrate technology into education and incorporate a technological approach. The International Society for Technology in Education (ISTE) developed a framework for teacher competence by focusing on students’ twenty first-century skills. Furthermore, it aims to aid teachers in their practice with technology, enhance collaboration among learners, innovate teaching through technology, and foster autonomy among learners (Crompton, 2017; ISTE, 2018). The ISTE standards for educators are categorized into seven types of competencies that educators are required to achieve during their career. Educators should improve their competence in evaluation, facilitation, designation, collaboration, leadership, and citizenship and in learning to work well with students and guiding them within a technological environment. The framework refers to the necessary competencies for teachers and provides teachers with examples of each competence and the focal competencies tied to the issues of teaching and learning with technology. However, the standard continues to hold certain limitations because the competencies are separate, whereas the illustrative examples for each competence are general and descriptive instead of practical. In Africa, the ICT-enhanced Teacher Standards framework (UNESCO International Institute for Capacity Building in Africa, 2012) was designed for countries on that continent. It comprises interrelated dimensions of competent, twenty first-century teachers in engaging in instructional design, namely, promotion and motivation of student learning, innovation and creation, creation and management of effective learning environments, evaluation and communication, professional development and model ethical duty, and comprehensive understanding of the subject. Additionally, various studies have used the frameworks noted above to assess teacher digital competence (e.g., Quaicoe & Pata, 2020), and the comprehensive subcomponents of teacher digital literacy and the relationship among them may be the reasons for the popularity of the framework.

Some nations adapted international frameworks to design and apply the national model to their specific educational context. For instance, the Spanish Ministry of Education, Culture and Sport designed a digital competence model in 2012 called the Common Framework for Digital Competence for Teachers (INTEF – Instituto Nacional de Tecnologías Educativas y Formación del Profesorado, 2017). It was adapted from DigComp (Carretero et al., 2017) and DigCompEdu (Redecker, 2017b) for the Spanish context. Eventually, several researchers used the framework to develop and validate instruments for measuring digital literacy (e.g., Tourón et al., 2018). Likewise, the Education and Teaching Foundation in Britain designed the Digital Teaching Professional Framework (Education & Training Foundation, 2019) to support educators in understanding technology integration, in cultivating the practice of teaching with technology, and in supplementing their professional development. The framework specifies teacher competence from seven aspects (planning pedagogy, approach pedagogy, student employability, specific teaching, assessment, accessibility and inclusion, and personal development). Each aspect is classified into three levels: exploration, adaptation, and leadership. As regards the seven aspects, the nature of each aspect was relatively consistent with a few international frameworks, such as DigCompEdu and UNESCO ICT-CFT. Moreover, the framework highlighted that the focal point of the competence of educators is to foster learners in using digital technology to enhance their employment and entrepreneurial prospects.

Multiple models and frameworks from various empirical studies have also contributed to the evaluation of digital literacy in specific educational contexts (e.g., Mishra & Koehler, 2006; Puentedura, 2006). Certain models or frameworks emphasize teacher competence in incorporating technology into the teaching process to enhance learning. Mishra and Koehler (2006) have developed a technological pedagogical content knowledge (TPACK) model that aims to illustrate effective teaching using technology to incorporate technological, pedagogical, and content knowledge. Undeniably, the framework has greatly contributed to integrating technology into the classroom. However, the model is limited because it does not offer a conceptualization of digital literacy for educators. Additionally, scholars have demonstrated the lack of discrimination between different areas of knowledge, while the boundaries of the aspects are vague (Drummond & Sweeney, 2017). Alternatively, the Substitution Augmentation Modification Redefinition (SAMR; Puentedura, 2006) model was developed as a descriptive framework with four levels of hierarchy (substitution, augmentation, modification, and redefinition). It follows the taxonomy from low to high levels and has been widely used by educational researchers and trainers as a guide for teachers in the technology integration process. At the highest level or stage (redefinition), teachers are guided to create new tasks that require abilities related to higher-order cognition, whereas at the starting stage (substitution), digital technology also functions as a guided tool that does not call for any change in the function of technology or created tasks with lower-order cognition. The two other intermediate steps, augmentation and modification, serve as bridges for transformation from the simple stage in using digital technologies to the more complex stage. These stages intend to facilitate development and innovation in education, pedagogy, and the curriculum. Although the SAMR model provided educators with a step-by-step process for achieving the target points in applying technology to the teaching process, it drew criticism because it lacked a detailed practical application and because it failed to specify the digital competence required of teachers for each stage and the transition from one stage to the next. Krumsvik (2014) introduced a digital competence model for teachers, which was developed in the Scandinavian context. The model grouped teacher digital competence into four subscales, basic technological usage, pedagogical use of digital technology, learning technology, and ethical issues, to align technology with education as well as to enhance awareness of digitization. It is evident that the model focused a great deal on evaluating a teacher’s competence in teaching with technology from the teacher’s perspective but not in relation to learners. Fisher et al. (2012) designed the teacher-centered DECK framework, which stresses the use of digital applications in teaching practice from four main aspects, distributed cognition and knowledge, engagement and motivation, community and communication, and knowledge enhancement. Although the framework clearly refers to digital literacy in practice, it does not provide adequate, detailed information on the competencies for each aspect. Hall et al. (2014) introduced a self-evaluation DigiLit Leicester framework that focuses on measuring digital literacy from four aspects with four levels (entry, core, developer, and pioneer), which were critically reviewed and adapted from different frameworks: (1) finding, assessment, and organization; (2) creation and sharing; (3) communication, cooperation, and participation; and (4) online safety and e-identity. Further, a number of frameworks concentrate on evaluating digital competence among pre-service teachers, with the components of these frameworks also being similar to those designed for inservice teachers. For instance, Expertise NetWork at the Ghent University Association (ENW AUGent, 2013) for teacher training institutions developed an ICT competence framework to support teacher training programs. The framework aimed to improve digital competence among preservice teachers in three broad dimensions of professional development, instruction and pedagogy, professional development, and the school. Table 1 lists typical but impressive frameworks in the field of educational digital literacy. One acceptable notion is that a number of competencies referred to by different frameworks and models at the cross-national, national, and contextual levels can be mapped using DigCompEdu (Redecker, 2017b). The reason is that the framework was evaluated to cover the main digital competencies of educators in the school context. Additionally, DigCompEdu was selected because it provided general competencies needed by educators to achieve digital literacy. Table 1 collates aspects of typical frameworks according to DigCompEdu.

**Table 1:** Teacher digital literacy frameworks

Framework/Model	Organization/Author	Country	Level	Component/Aspect	PE	DR	TL	A	EL	FLC
DigCompEdu	European Commission; Redecker (2017b)	International	Six levels: awareness, exploration, integration, expertise, leadership, and innovation	Professional engagement (PE), digital resources (DR), teaching and learning (TL), assessment (A), empowering learners (EL), and facilitating learners’ digital competence (FLC)	O	O	O	O	O	O
ICT Competency Framework for Teachers	UNESCO	International	Three levels: knowledge acquisition, knowledge deepening, and knowledge creation	Policy and vision, curriculum and assessment, pedagogy, ICT, organization and administration, and teacher professional development	O	O	O	O	O	O
ISTE	Crompton (2017)	USA		Evaluation, facilitation, designation, collaboration, leadership, citizenship, and learning	O	O	O	O	O	O
The Common Framework for Digital Competence for Teachers	The Spanish Ministry of Education, Culture and Sports	Spain	Three levels: basic, medium, and advanced	Information and data literacy communication and collaboration, digital content creation, safety, problem-solving	O	O	O		O	O
The British Framework of Digital Teaching	The British Education and Teaching Foundation	Britain		Pedagogical planning, pedagogical approach, employability of students, specific teaching, evaluation, accessibility and inclusion, and self-development	O	O	O	O	O	O
Teachers’ ICT competencies	Ministry of Education, Chile; Enlances (2011)	Chile		Pedagogical, technical, management, social, ethical, legal, and professional development	O	O	O			
DigEuLit	Martin and Grudziecki (2006)	Contextual	Three levels: digital competence, digital usage, and digital transformation	Skills/concepts, professional application, and innovation	O	O	O			
SARM	Puentedura (2006)	Contextual	Four levels: substitution, augmentation, modification, and redefinition	Visualization and simulation, social computing, digital storytelling, and educational gaming	O	O	O			O
TPACK	Mishra and Koehler (2006)	Contextual		Technology, pedagogy, content, and knowledge	O		O			O
Teachers’ digital competence model	Krumsvik (2014)	Contextual	Four levels: adoption, adaptation, appropriation, innovation	Basic digital skills, elementary skills, didactic ICT competence, learning strategies, and digital building	O	O	O		O	
DECK	Fisher et al. (2012)	Contextual		Distributed cognition and knowledge, engagement and motivation, community and communication, and knowledge building	O	O				O
DigiLit Leicester	Hall et al. (2014)	Contextual	Four levels: entry, core, developer, pioneer	Finding, evaluating, and organizing; creating and sharing; communication, collaboration, and participation; and e-safety and online identity	O	O	O			O
Digital Literacy Model	Ng (2012)	Contextual		Technical, cognitive, and socioemotional	O	O	O			O

### Approaches and types of instruments to measure digital literacy

Two approaches for measurement (pragmatic and psychometric) are common in designing tools to assess digital literacy. Each approach has its strengths and weaknesses as regards validity. Scholars have thus advised combining the two approaches to guarantee instrument validity. Based on multiple frameworks and models, international and national organizations and researchers in specific contexts have designed and improved various tools to assess digital literacy. Assessment tools can measure information, technology, and digital information and are thus grouped from the perspective of assessment and item design or based on their objectives (e.g., research purpose and quality insurance; Sparks et al., 2016). The most common means of classifying digital literacy instruments is the use of the data collection approach, which includes knowledge-based assessment (response on the manner of handling tasks), performance assessment (illustration of the manner of performing tasks), and self-assessment (self-evaluation of the competence of completing tasks; Carretero et al., 2017). Obtaining a large number of participants is seemingly difficult for performance and knowledge assessment in contrast to self-assessment. However, implementing tools for self-evaluation research may result in low reliability and validity. Also, multiple researchers have indicated that there is a low correlation between students’ self-reported digital literacy and their actual performance (Hatlevik et al., 2018), and the result of this indirect assessment only reports a belief about digital literacy. Therefore, when designing a digital literacy assessment tool, there has been an attempt to immerse instruments in an authentic digital environment (Reichert et al., 2020). However, there is also a concern that teachers or students may not have the necessary technical/operational competence to use the assessment tool (Chanta, 2021), and the poor result of the level of digital literacy might not be a result of participants’ actual performance but their lack of digital competence in using the assessment software. This means that there is no perfect digital literacy assessment tool, and it is always a challenging task to measure teachers’ or students’ digital literacy. This is why scholars need to consider a number of factors when designing an instrument, such as the research context, participants, facilities, and so on. Additionally, technologies are developing by the minute, so digital literacy assessment instruments need updating to keep pace with the new technologies.

### Instrument reliability and validity indicators

The reliability of a measurement depends on the degree to which it provides a stable and consistent result (Carmines & Zeller, 1979), whereas validity denotes the ability to measure what one intends to measure (Field, 2005). In terms of reliability, Cronbach’s alpha coefficient is the most common indicator to measure the internal consistency of the assessment tool. As regards validity, scholars have reported on the common types of validity in the development and validation of instruments, such as face, content, construct, and so on. Face validity refers to researchers’ subjective evaluation of the relevance, clarity, and rationality of the instrument (Oluwatayo, 2012), and Cohen’s Kappa Index (CKI) is normally used to determine the face validity of the assessment tool. Content validity refers to the extent to which items in an instrument measure how comprehensive and representative the content of the instrument is (Newman et al., 2006). Scholars commonly use a content validity index, which can be calculated with several methods to prove the validity tied to the content of the instrument. In addition to face and content validity, construct validity, which refers to the extent to which the assessment instrument really evaluates what it purports to (Ginty, 2013), also needs to be reported for instrument quality assurance. The validity of the construct is guaranteed through two subsets: convergent validity and discriminant validity.

The current study investigates the extent to which an instrument for measuring digital literacy is reliable and valid by determining the types of reliability and validity that researchers used to validate the instruments.

## Method

### Search strategies and study selection

**Fig. 1:**
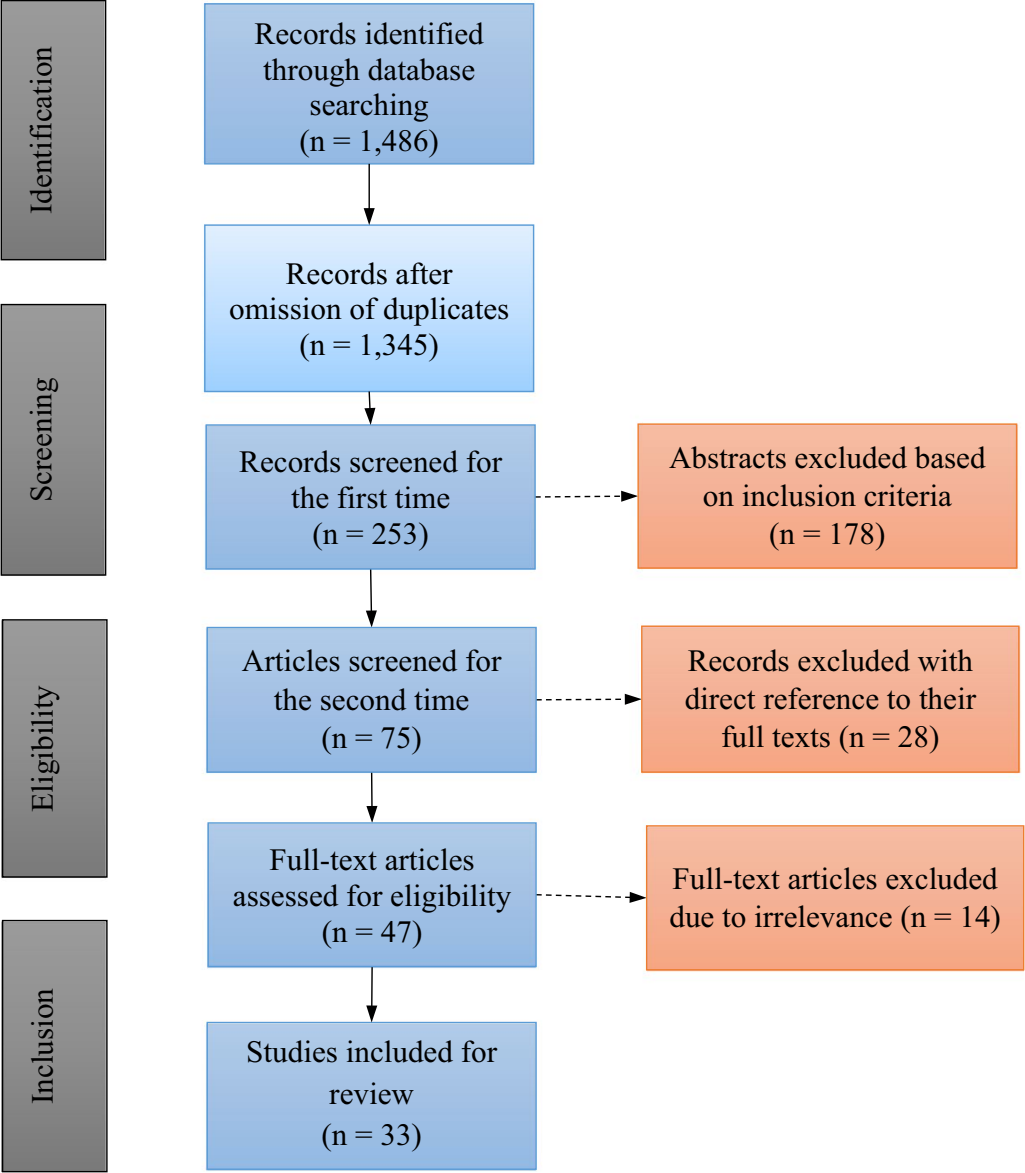
Process of selecting articles for review (adapted from the PRISMA flow diagram)

A search was conducted in three scientific databases (Education Resources Information Center (ERIC), Web of Science, and Scopus) and was based on the Preferred Reporting Items for Systematic Reviews and Meta-Analyses (PRISMA) flow diagram (Fig. 1; Moher et al., 2009). We conducted electronic and manual searches to identify target articles. Papers were selected for review on the basis of five major inclusion criteria:

- Articles were limited to peer-reviewed journals between January 2011 and February 2022.
- Articles were published on information technology research in education.
- The study participants were in service and pre-service teachers.
- The study focused on digital competency assessment.
- The articles were written in English.

Articles were searched for with the following search terms in the title, keywords, and abstracts: “ICT competency” or “ICT literacy” or “digital literacy” or “information literacy” or “computer literacy” or “technology literacy” and “assessment.” The second to fifth listed here were included because they are subfields of ICT competency (Tristán-López & Ylizaliturri-Salcedo, 2014).

Following the search strategy, the study identified 1486 articles in the databases noted above. After we omitted duplicates and screened the abstracts and full texts based on the inclusion criteria, the articles were placed on a list for further review to guarantee that the articles selected met the requirements. Moreover, they were reviewed to search for answers to the research questions. Finally, 33 studies, which had been published in high-quality, peer-reviewed journals to ensure the reliability and validity of the research, were selected for the review.

### The coding process and data extraction

After finalizing the studies eligible for review, we coded 33 studies for features, as described in Table 2. We used the code to specify the research context and identify information tied to components of digital literacy assessment tools, types of instruments, evidence for reliability and validity, and frameworks or models used to design the assessment tool in each study. The authors coded the features of all the studies selected, especially collating the aspects of digital literacy components which were used in each assessment tool according to the DigCompEdu framework. The data extraction was then reviewed and revised multiple times with the agreement of the authors before it was synthesized and interpreted for the results and discussion.

**Table 2:** Coding scheme for studies selected

Study coding	
Research context	Year, author, country, level of schools
Sample	Number of pre-service or inservice teachers in the study
Research tools	Questionnaire, interview, performance task, etc.
Types of assessment tool	Self-evaluation, objective assessment, etc.
Framework/model	Framework or model used in designing the assessment tool
Components of digital literacy assessment tool	Professional engagement, digital resources, teaching and learning, assessment, empowering learners, and facilitating learners’ digital competence
Evidence of reliability and validity	Reliability (Cronbach’s α, kappa coefficient, McDonald’s omega, etc.)
	Validity (content validity, face validity, construct validity, etc.)

## Results and discussion

### Characteristics of reviewed studies

**Fig. 2:**
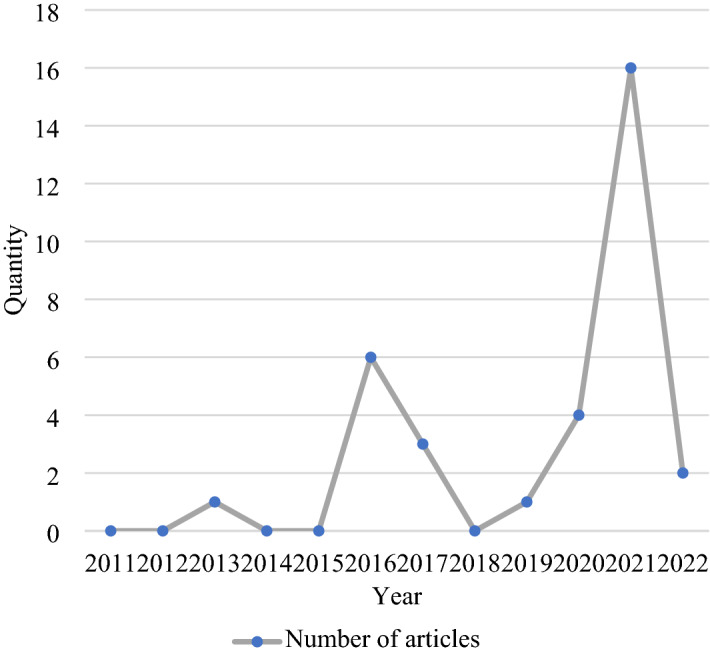
Distribution of articles per year

Among the selected papers, 15 (44%) were conducted to determine the level of digital competence among pre-service teachers, whereas 18 (55%) explored that of inservice teachers at primary and secondary schools, at universities, and in other areas (e.g., vocational education and training and special education) (50%, 55%, 22%, 22%, and 11%, respectively). Figure 2 and Table 3 depict the distribution of the selected articles per year and per peer-reviewed journal, respectively.

**Table 3:** Distribution of selected articles per journal

Journal title	Number of articles
Computers and education	4
Nordic journal of digital literacy	2
Computers in human behavior	2
British journal of educational technology	2
Education sciences	5
Journal of new approaches in educational research	3
System	1
Educacion XX1	1
Education and information technologies	2
Journal of education for teaching	1
European journal of teacher education	1
Journal of information and technology education: research	1
International education studies	1
Journal of educational computing research	1
European journal of contemporary education	1
Empirical research in vocational education and training	1
Scandinavian journal of educational research	1
Technology, knowledge and learning	1
Asia pacific journal of educators and education	1
Interactive learning environments	1

### RQ1: what aspects of digital literacy are presented in recent research on potential ways to assess teacher digital literacy?

**Fig. 3:**
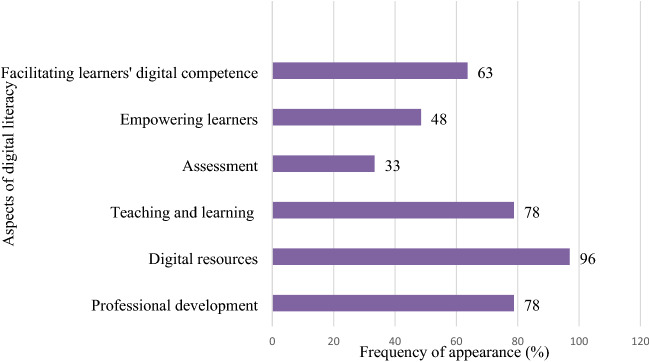
Frequency of appearance of the main aspects of digital literacy in reviewed papers

The studies selected used multiple instruments to examine different aspects based on various frameworks to identify the level of teacher digital competence. We categorized these aspects on the basis of the six main components of the DigCompEdu framework. The result demonstrates that among the aspects of digital literacy cited, the papers reviewed exhibited a trend of mainly focusing on exploring teacher competence in integrating digital resources (97%), teaching and learning (78%), improving professional development (78%), and facilitating learner digital competence (63%). The other two teacher competencies of assessment and empowering learners are less investigated (33% and 48%, respectively). Figure 3 presents a comparison of the frequency of appearance of the six main aspects of digital literacy in published articles.

Out of the 33 selected papers, ten investigated teacher competence within the six elements of digital literacy (e.g., Çebi & Reisoğlu, 2022). In other studies, researchers only highlighted a few major aspects, which are considered the core competencies that influence teaching and learning effectiveness. For instance, Wong and Moorhouse (2021) endeavored to assess digital competence among English as a foreign language (EFL) teachers in primary and secondary schools in Hong Kong in terms of applying educational technologies, teaching and learning, evaluation, and empowering students during the forced transition of schools to online education due to the COVID-19 pandemic. These four interconnected competencies are subcomponents of educators’ pedagogical competencies based on the DigCompEdu framework (Redecker, 2017b). Meanwhile, other studies were conducted to examine only one aspect of teacher digital literacy in depth. For example, Potyrała and Tomczyk (2021) explored teacher knowledge and skills when addressing online safety, such as the evaluation of the reliability of e-information, cyberbullying, and other issues related to the application of technology to education. A few scholars also explored teacher competence in using new aspects apart from the six core components. Alarcón et al. (2020) contributed to the DigCompEdu framework by adding two components, digital environment and extrinsic digital engagement, which are external components that influence digital literacy. The questionnaire thus developed proved reliable and valid according to the construct validity and psychometric properties of the Spanish educational context.

### RQ2: what types of instruments are used to assess digital literacy, and how are instrument reliability and validity reported?

The literature has designed, developed, or adapted various instruments to determine teacher digital literacy. In the papers reviewed, the researchers used diverse assessment instruments, which can be categorized into self-evaluation, objective evaluation (knowledge- or performance-based assessment), and a combination of the two (85%, 6%, and 9%, respectively).

In total, 28 papers used only self-assessment when exploring teacher digital competence. The reason for the popularity of self-assessment is that data collection from a large number of participants is convenient and economical. Additionally, self-assessment may be useful in eliciting teacher reflection on their knowledge, skills, and attitudes toward the use of educational technologies in teaching. However, the general limitation of these studies is that they only use self-assessment to evaluate competencies in the use of technology in teaching because the literature has demonstrated that certain groups of participants tend to overestimate or underestimate their competence compared with their actual level. This aspect may lead to inadequate knowledge and skills in the management of technologies and media in teaching.

Thus, the use of self-assessment questionnaires may not achieve adequate reliability and internal validity because of the limitations noted above. For this reason, a few authors in recent years have tended to use more than one instrument by adding other objective tools for evaluation when assessing teacher digital literacy to reinforce the persuasiveness of the findings. For example, Nebot et al. (2021) conducted a mixed-method study and explored digital competence among university instructors with a self-assessment questionnaire followed by interviews with the objective of presenting rich descriptions using quantitative and qualitative data. A holistic understanding of the target issue was thus achieved. Maderick et al. (2016) also included objective assessment in conjunction with self-assessment to examine teacher digital competence. This group of authors used two types of questionnaires (Likert and multiple-choice scales for knowledge-based assessment) to evaluate competence subjectively and objectively in parallel. A few studies only used objective assessment through knowledge tests. For example, Wang and Lu (2021) developed items with multiple types of modalities, such as multiple-choice, true or false, and fill in the blanks, for an objective assessment of technological competency among pre-service teachers in China. Additionally, the participants were supported with text, videos, or images during the test. The majority of the studies selected did not conduct performance-based assessment using performance tests but observations, such as Røkenes and Krumsvik’s mixed-method research (2016). Specifically, the researchers observed pre-service teachers to determine how they used technology in the language classroom. They found that digital literacy has received increased scholarly attention in recent years due to the emergence of hybrid educational environments. Moreover, teacher digital literacy is considered the principal element in the effectiveness of technological innovation. Thus, the results suggest that researchers should use additional and other reliable and valid methods to assess digital literacy objectively through the support of technology or authentic and interactive tests. In this manner, pre-service and inservice teachers become aware of their actual levels of competence in technology. As such, teacher training programs can be adjusted to aid pre-service teachers in building the foundation of their competence and, therefore, to improve it, especially in using technology applications to meet the demands of the future teaching environment. Moreover, workshops, seminars, or short training courses can be designed for inservice teachers to fill the research gap in terms of the effectiveness of managing educational tools in the classroom.

In terms of the reliability and validity of the assessment tools, critical to determining the efficacy of the research tools, 26 out of 33 papers (78%) reported reliability, 22 (66%) presented validity values, and 21 (63%) pointed out the levels of instrument reliability and validity. The results demonstrate that researchers generally describe the level of instrument reliability with Cronbach’s alpha (α) coefficient (e.g., Lucas et al., 2021; Quaicoe & Pata, 2020). Frequently, reliability is evaluated using the omega (Rubach & Lazarides, 2021) or kappa (Cantabrana et al., 2019) coefficient or expected a posteriori/plausible values (Wang & Lu, 2021). A few papers partially reported evidence of instrument validity (Wong & Moorhouse, 2021), whereas others reported that the instruments are reliable and valid for the research context (e.g., Teo et al., 2016; Tondeur et al., 2017; Wang & Lu, 2021). In the case of instrument validity, except for content, face, convergent, discriminant, and construct validity, several scholars also analyzed goodness-of-fit at the item level. Out of the papers reviewed, Wang and Lu (2021) confirmed item validity by demonstrating the fit of an item with the participant. By analyzing the goodness-of-fit of various items and levels of difficulty, the researchers differentiated examinees’ skills and level of knowledge. Whether a paper partially or fully describes instrument reliability and validity depends on the objective of the study. Nevertheless, the basic values of reliability and validity should be reported to enhance the persuasiveness of the findings.

### RQ3: what frameworks or models are used to develop the instruments?

In the literature, various frameworks and models have been developed at the international, national, and contextual levels to evaluate teacher digital literacy. Among the reviewed papers, instruments were designed, developed, and adapted on the basis of such frameworks. Among the publications selected, seven (21%) used research tools designed and developed based on the DigCompEdu framework, which includes educators’ general and necessary competencies. Although the framework was designed for the educational context of European countries, it has been applied to other environments (e.g., Alarcón et al., 2020; Karunaweera & Wah, 2021). For instance, Alarcón et al. (2020) extended DigCompEdu by adding two components to enable the questionnaire to match the specific context of technology use. The research tool was later used to collect data from participants in a European country (Spain) and countries in Latin America, such as Mexico, Chile, and Peru. DigCompEdu was developed on the basis of DigComp to evaluate digital competence in education; however, a few researchers continue to refer to the DigComp framework when producing questionnaires intended to assess the competence of educators as digital citizens of the twenty-first century. In certain cases, DigComp has been combined with other digital models for education, such as that of Rubach and Lazarides (2021), to examine teachers’ competence in applying technology to teaching practice. Out of the papers selected, only one involved UNESCO ICT-CFT, which was used as a scaffold to measure e-skills among university teaching staff in Ecuador (Jorge-Vázquez et al., 2021). These results demonstrate that studies on digital literacy in relation to infrastructure and policy are scarce, whereas the enhancement of digital competence may be influenced by facilities and strategic leadership (Wastiau et al., 2013). Other studies have cited other international frameworks, such as the ICT-Enhanced Teacher Standards for Africa (Quaicoe & Pata, 2020), and other national frameworks constructed on the basis of the international frameworks (e.g., Prieto-Ballester et al., 2021; Wang & Lu, 2021). Still other studies have used models developed from specific contexts such that the competencies fit the educational environment (e.g., Cantabrana et al., 2019). In total, seven studies developed research tools based on the theories or definitions in the literature (e.g., Hatlevik, 2016) or focused on evaluating teachers’ knowledge of digital tools (e.g., Maderick et al., 2016) or competency in managing and processing various types of educational technologies (e.g., Záhorec et al., 2021). Although various frameworks are available at different levels, no fixed frameworks or models that fit all contexts exist, whereas multiple types of research tools have been used to measure teacher digital literacy. Thus, teachers are required to achieve updated technological competence and other related knowledge and skills to enable them to keep pace with society and to aid students in becoming digitally competent citizens of the technology era due to the unprecedented development in technology and media.

## Conclusions, limitations, and recommendations

The literature review was conducted to enhance our understanding of how researchers assess teacher digital literacy. By reviewing 33 papers published in peer-reviewed journals and in educational technology, we address the major aspects of digital literacy generally explored by researchers, various forms of research tools used to assess digital literacy, and the common frameworks and models used as a scaffold to develop research tools. Various aspects of digital literacy exist in the digital competence framework, models, and instruments. Thus, the researchers categorized these aspects on the basis of the six main components of DigCompEdu, which is one of the most influential frameworks. The findings show that the majority of researchers emphasize the assessment of teacher digital competence from four aspects in improving their digital competence: using various educational technologies, teaching and learning, professional development, and supporting learners. However, only a few studies highlighted the manner in which teachers use technology to assess students’ learning outcomes and how they empower learners to be in charge of the e-learning process. Also, digital competence should be discussed in the light of technological and educational environments with the support of leadership, facilities, and policy. Among the studies selected, only one explored these issues, while leadership, facilities, and policy have considerable impacts on enhancing digital competence. Moreover, self-evaluation instruments are relatively common research tools in the publications selected, whereas a number of papers use solely subjective assessment. A few papers addressed the weaknesses of subjective evaluation by combining different types of instruments to assess teacher digital literacy. Moreover, studies partially or fully reported the reliability and validity of multiple instruments according to the objective of the research. Additionally, the findings demonstrate that DigCompEdu and DigComp are the two most common frameworks used to develop instruments to assess teacher digital competence. The two frameworks also form the foundation for the design of other frameworks at the national and contextual levels.

The paper provides a picture of research tools used to assess teacher digital literacy in the school context. The articles were selected after conducting a search in three databases (ERIC, Web of Science, and Scopus) from 2011 to 2022. The current study only includes papers that were searched for in these databases, and only papers published in English were selected. Hence, other articles on the topic may have been written in other languages but have been omitted. Future research may expand the search areas and review publications in other languages to depict the full picture of the assessment of teacher digital competence. The findings indicate that numerous studies have assessed teacher digital literacy using self-assessment tools. Thus, future research may develop and design other authentic and interactive tests to reflect teachers’ actual knowledge and skills in terms of technology integration. Additionally, a few papers used the mixed-method approach to evaluate target competencies. Therefore, further studies that combine quantitative and qualitative methods should be conducted to provide a comprehensive understanding of teacher digital competence in specific contexts. Additionally, subjective and objective evaluations should be incorporated into these tools to reflect the exact level at which teachers apply technology to education. Furthermore, the findings demonstrate that the percentage of papers that focus on examining teacher competence in terms of using educational technology for assessment and empowering learners remains low. Thus, the need emerges to focus on these two aspects of digital literacy when assessing teacher digital competence. Moreover, the results show that a large number of studies concentrate on evaluating teacher digital competence in primary and secondary schools. In this case, the context of technology integration among teachers in high school, higher education, and other educational contexts (e.g., vocational and special schools) warrants further exploration.

## Appendix

See Table

**Table 4:** Overview of instruments for assessing teachers’ digital literacy

No	Author (Year)	Country	Sample size	Level of schools	Methodology	Research Tool	Type	Framework/Model	Aspects	Reliability evidence	Validity evidence
1	Wong and Moorhouse (2021)	Hongkong	73	Primary Secondary	Mix-methods	Open-ended questionnaire Semi-structured interviews	Self-evaluation	DigCompEdu	Digital resources, teaching and learning, assessment, empowering learners		Content and face validity
2	Rodríguez et al. (2021)	Spain	144	Pre-service teachers)	Quantitative	Likert Scale questionnaire	Self-evaluation		Didactic, curricular and methodological aspects, planning, organization and management of digital technological resources and spaces, ethical, legal and security aspects, personal and professional development	Cronbach’s α	Content validity Construct validity (exploratory factor analysis)
3	Røkenes and Krumsvik (2016)	Norway	270	Pre-service teachers)	Mix-methods	Observation notes Likert scale questionnaire Semi Structured interview	Self-evaluation	Krumsvik (2014)‘s digital competence model	Basic digital skills, didactic ICT competence, learning strategies, digital Bildung	Cronbach’s α	Construct validity (exploratory factor analysis, confirmatory factor analysis)
4	Lucas et al. (2021)	Portugal	1071	Primary school Secondary	Quantitative	Likert scale questionnaire	Self-evaluation	DigCompEdu	Professional Engagement, digital resources, teaching and learning, assessment, empowering learners, facilitating learners’ digital competence	Cronbach’s α	Content validity Construct validity
5	Cattaneo et al. (2022)	Switzerland	1692	Vocational education and training	Quantitative	Likert scale questionnaire	Self-evaluation	DigCompEdu	Communication and collaboration, professional development, digital resources selection,digital resources creation,data protection, teaching and learning, assessment, learners’ empowerment, media education, learners digital competence	Cronbach’s α	Construct validity (confirmatory factor analysis)
6	Rubach and Lazarides (2021)	Germany	372	Primary Secondary	Quantitative	Likert scale questionnaire	Self-evaluation	DigComp teachers’ basic ICT competencies (Tourón et al., 2018) Basic ICT digital competence (Kultusministerkonferenz, 2016)	Information and data literacy, communication and collaboration, digital content creation, safety and security, problem-solving, analyzing and reflecting	Cronbach’s α	Construct validity (exploratory factor analysis, confirmatory factor analysis)
7	Potyrała and Tomczyk (2021)	Poland	484	Lower secondary	Quantitative	Likert scale questionnaire Multiple choice questionnaire	Self-evaluation Objective evaluation		Online safety	Cronbach’s α	Content validity Construct validity (exploratory factor analysis)
8	Casillas Martín et al. (2020)	Spain	332	Pre-service teachers	Quantitative	Likert scale questionnaire	Self-evaluation	Teachers’ digital competence model	Basic digital competences, teaching approach competence in the use of learning strategies	Cronbach’s α	Content validity
9	Peled (2021)	Israel	1265	Pre-service teachers	Quantitative	Likert scale questionnaire	Self-evaluation	The seven domains of digital literacy Kurtz and Peled (2016)	Information collection, information evaluation, information management, information processing, teamwork, integrity awareness, and social responsibility	Cronbach’s α	
10	Danner and Pessu (2013)	Nigeria	100	Pre-service teachers	Quantitative	Likert scale questionnaire	Self-evaluation		Digital resources	Cronbach’s α	
11	Gutiérrez Porlán and Serrano Sánchez (2016)	Spain	134	Pre-service teachers	Quantitative	Likert scale questionnaire	Self-evaluation	DigComp	Information, communication, creation of content, safety, problem-solving		
12	Al Khateeb (2017)	Saudi Arabia	108	Primary Secondary High school	Quantitative	Likert scale questionnaire	Self-evaluation	DigComp	Information processing, communication control, creation, safety, problem-solving	Cronbach’s α	Construct validity (exploratory factor analysis)
13	Maderick et al. (2016)	USA	174	Pre-service teachers	Quantitative	Likert scale questionnaire Multiple choice questionnaire	Self-evaluation Objective evaluation		Digital resources	Cronbach’s α	Construct validity (confirmatory factor analysis)
14	Alarcón et al. (2020)	Spain Mexico Chile Peru Ecuador Venezuela Argentina Colombia	509	University	Quantitative	Likert scale questionnaire	Self-evaluation	Extended DigCompEdu	Professional engagement, digital resources, teaching and learning, assessment, empowering learners, facilitating learners’ digital competence digital environment, extrinsic digital engagement	Cronbach’s α	Construct validity (confirmatory factor analysis)
15	Çebi and Reisoglu (2020)	Turkey	518	Pre-service teachers	Quantitative	Likert scale questionnaire	Self-evaluation	DigComp	Information and data literacy, communication and collaboration, digital content creation, safety, problem-solving		
16	Záhorec et al. (2021)	Slovakia	173	Primary Secondary	Quantitative	Likert scale questionnaire	Self-evaluation		Digital resources		
17	Tondeur et al. (2017)	Belgium	931	Pre-service teachers	Quantitative	Likert scale questionnaire	Self-evaluation	The ICT competence framework (ENW AUGent, 2013)	ICT competencies to support pupils for ICT use, ICT competencies for instructional design	Cronbach’s α	Content validity Construct validity (exploratory factor analysis, confirmatory factor analysis)
18	Cantabrana et al. (2019)	Spain	25	Pre-service teachers	Quantitative	Multiple choice questionnaire	Objective assessment	Lazaro Cantabrana and Gisbert Cervera (2015) Teacher digital competence rubric	Didactic, curricular and methodological; planning, organization and management of digital technological resources and spaces; relational, ethical and security; personal and professional development	Kappa coefficient	Content validity Construct validity
19	Quaicoe and Pata (2020)	Ghana	233	Primary Junior high school	Quantitative	Likert scale questionnaire	Self-evaluation	The ICT-enhanced teacher standards for Africa	Instructional design, facilitating and inspiring student learning, managing learning environment, learner assessment and communicating student learning, professional development, and subject area mastery	Cronbach’s α	
20	Roll and Ifenthaler (2021)	an Europe county	222	Pre-service teachers	Mixed method	Likert scale questionnaire Open-ended questionnaire	Self-evaluation	Combination of several frameworks (e.g., TPACK)	Attitude towards digitization, handling of digital devices, information literacy, application of digital security, collaboration due to digital communication, solving of digital problems, and reflection on the interconnected and digital environment	Cronbach’s α	Construct validity (confirmatory factor analysis)
21	Hatlevik (2016)	Norway	312	Primary Secondary	Quantitative	Likert scale questionnaire	Self-evaluation		Search and process, produce, digital responsibility, communication	Cronbach’s α	Construct validity (confirmatory factor analysis)
22	Wang and Lu (2021)	China	182	Pre-service teachers	Quantitative	Multiple choice, true/false, and fill-in-the-blank questionnaire	Objective evaluation	Chinese educational technology standard has been developed specifically for pre-service teachers	Basic technology competency, technology-supported learning, technology-supported teaching	Expected a posteriori/ plausible values	Content validity, Construct validity (convergent validity, discriminant validity, confirmatory factor analysis) Item validity
23	Ramírez-Montoya et al. (2017)	Not mentioned	863	Primary High school Tertiary Others	Mixed method	Likert scale questionnaire Open-ended questionnaire	Self-evaluation Report		Technical ability to use digital resources	Cronbach’s α	Content validity
24	Çebi and Reisoğlu (2022)	Turkey	697		Quantitative	Likert scale questionnaire	Self-evaluation	DigCompEdu	Professional engagement, digital Resources, teaching and learning, assessment, empowering learners, facilitating learners’ digital competence	Cronbach’s α	Construct validity (convergent validity, confirmatory factor analysis)
25	Bandrés et al. (2021)	Spain	121		Quantitative	Likert scale questionnaire	Self-evaluation		Professional engagement, digital resources, teaching and learning, assessment, empowering learners, facilitating learners’ digital competence		
26	Kotzebue et al. (2021)	Germany	118	Pre-service teachers	Quantitative	Likert scale questionnaire	Self-evaluation	DiKoLAN	Documentation, presentation, communication/collaboration, and information search, data acquisition, data processing, simulation and modeling	Cronbach’s α	Construct validity (path model)
27	Jorge-Vázquez et al. (2021)	Ecuador	216	University	Quantitative	Likert scale questionnaire	Self-evaluation	UNESCO ICT-CFT	Policy and vision, curriculum and assessment, pedagogy, ICT, organization and administration, teacher professional development	Cronbach’s α	
28	Tomczyk (2021)	Poland	128	Pre-service teachers	Quantitative	Survey questionnaire Test	Self-evaluation Objective evaluation		Digital resources	Cronbach’s α	Construct validity (exploratory factor analysis, confirmatory factor analysis)
29	Nebot et al. (2021)	Spain	61	University	Mixed method	Likert scale questionnaire Interview	Self-evaluation	DigCompEdu	Professional engagement, pedagogical competences, students’ competences	Cronbach’s α	Content validity
30	Karunaweera and Wah (2021)	Sri Lanka	40	Primary Secondary	Quantitative	Likert scale questionnaire	Self-evaluation	DigCompEdu	Professional engagement, digital resources, teaching and learning, assessment, empowering learners, facilitating learners’ digital competence		
31	Krumsvik et al. (2016)	Norway	2477	High school	Quantitative	Likert scale questionnaire	Self-evaluation	Teachers’ digital competence model (Krumsvik, 2014)	Elementary ICT, basic ICT skills, didactic ICT competence, digital learning strategies, digital Bildung	Cronbach’s α	
32	Teo et al. (2016)	Turkey	557	Pre-service teachers	Quantitative	Likert scale questionnaire	Self-evaluation	Digital native assessment scale (Teo, 2013)	Grow up with technology, are comfortable with multitasking, are reliant on graphics for communication, and thrive on instant gratifications and rewards	Composite reliability, average variance extracted	Face validity Content validity Construct validity (confirmatory factor analysis)
33	Prieto-Ballester et al. (2021)	Spain	208	Secondary	Mixed method	Likert scale questionnaire Open-ended questionnaire	Self-evaluation	INTEF	Information, communication, content creation, security, and problem-solving		

4.
